# Systems genomics study reveals expression quantitative trait loci, regulator genes and pathways associated with boar taint in pigs

**DOI:** 10.1371/journal.pone.0192673

**Published:** 2018-02-13

**Authors:** Markus Drag, Mathias B. Hansen, Haja N. Kadarmideen

**Affiliations:** 1 Section of Anatomy, Biochemistry and Physiology, Department of Veterinary and Animal Sciences, Faculty of Health and Medical Sciences, University of Copenhagen, Frederiksberg C, Denmark; 2 Section of Systems Genomics, Department of Bio and Health Informatics, Technical University of Denmark, Kemitorvet, Lyngby, Denmark; China Agricultural University, CHINA

## Abstract

Boar taint is an offensive odour and/or taste from a proportion of non-castrated male pigs caused by skatole and androstenone accumulation during sexual maturity. Castration is widely used to avoid boar taint but is currently under debate because of animal welfare concerns. This study aimed to identify expression quantitative trait loci (eQTLs) with potential effects on boar taint compounds to improve breeding possibilities for reduced boar taint. Danish Landrace male boars with low, medium and high genetic merit for skatole and human nose score (HNS) were slaughtered at ~100 kg. Gene expression profiles were obtained by RNA-Seq, and genotype data were obtained by an Illumina 60K Porcine SNP chip. Following quality control and filtering, 10,545 and 12,731 genes from liver and testis were included in the eQTL analysis, together with 20,827 SNP variants. A total of 205 and 109 single-tissue eQTLs associated with 102 and 58 unique genes were identified in liver and testis, respectively. By employing a multivariate Bayesian hierarchical model, 26 eQTLs were identified as significant multi-tissue eQTLs. The highest densities of eQTLs were found on pig chromosomes SSC12, SSC1, SSC13, SSC9 and SSC14. Functional characterisation of eQTLs revealed functions within regulation of androgen and the intracellular steroid hormone receptor signalling pathway and of xenobiotic metabolism by cytochrome P450 system and cellular response to oestradiol. A QTL enrichment test revealed 89 QTL traits curated by the Animal Genome PigQTL database to be significantly overlapped by the genomic coordinates of *cis*-acting eQTLs. Finally, a subset of 35 *cis*-acting eQTLs overlapped with known boar taint QTL traits. These eQTLs could be useful in the development of a DNA test for boar taint but careful monitoring of other overlapping QTL traits should be performed to avoid any negative consequences of selection.

## Introduction

Boar taint is an offensive odour and/or taste observed in cooked meat from a proportion of non-castrated male pigs undergoing sexual maturity and is primarily related to two compounds: skatole (3-methylindole) from the hindgut and androstenone (5α-androst-16-ene-3-one) from the testis [1]. The compounds accumulate in adipose tissue and are correlated with sexual maturity at the time of slaughter [2, 3] and/or the amount of liver degradation [4, 5]. In the liver, a high concentration of androstenone prevents breakdown of skatole by inhibiting enzymes responsible for skatole metabolism [6]. Furthermore, androstenone itself causes boar taint, and previous research has shown that the compound is primary in the hierarchy of boar taint development [7]. To avoid boar taint, castration is usually performed at an early age, but this strategy is under debate because of animal welfare concerns [8]. As an alternative to castration, gene-based selection [9] through large-scale breeding programmes using predictive biomarkers for reduced boar taint in non-castrated male pigs has been proposed [10–15]. Skatole and androstenone are moderate to highly heritable traits [16–18] depending on the breed [4], and previous work has established low and mostly favourable genetic correlations between boar taint compounds and production traits such as meat quality [19]. Furthermore, selection could be performed from birth in both sexes, not only after sexual maturity [20], which could accelerate genetic gain [21]. Hence, gene-based selection should be possible without negatively affecting important male fertility traits [19] or sexual maturation [6].

To accelerate genetic control of boar taint and other economically important traits, vast numbers of quantitative trait loci (QTLs) have been identified [4]. Currently, the Pig Quantitative Trait Locus (QTL) Database (PigQTLdb) [22] has information on 25,473 QTLs from 593 publications that represent 646 different traits (as of December 2017). Importantly, QTLs may provide information on genes that could affect phenotype (positional candidate gene approach) [23]. Most of the published data derive from studies involving experimental crosses using pig breeds exhibiting extremes for the phenotypes of interest [24]. Previous studies on QTLs for levels of skatole, androstenone and indole have shown that these traits are highly polygenic [10, 11, 21, 25], and efforts to increase precision and accuracy have led to complementation of QTL studies by genome-wide association studies (GWAS) through high-density single-nucleotide polymorphism (SNP) panels. These studies are usually performed with Illumina Porcine SNP60 Genotyping BeadChips, which allow for the identification of significant association with the phenotype of interest. In contrast to traditional QTL mapping, GWAS provides unlinked individual genes or SNPs, which are easy to separate for analysis, but the caveat may be a large number of false positives [26]. Previous research involving one thousand Danish Landrace boars with divergent levels of skatole and androstenone identified a number of associated SNPs for skatole on pig chromosome (SSC) 14 and for androstenone on SSC5 [15]. A number of other breeds have been subjected to GWAS for SNPs associated with androstenone and/or skatole levels, including Pietrain [27], Duroc and Norwegian Landrace [12].

In recent years, extensive efforts have been made to incorporate next-generation sequencing into the elucidation of the genetic determinants of economically important traits. These new technologies, such as RNA sequencing (RNA-Seq), provide an unprecedented level of accuracy and precision for measurements of gene expression profiles (transcriptome) [28]. To accommodate new technologies into the framework of genomics and breeding development, the concept of “systems genomics” was introduced by Kadarmideen, von Rohr [29]. The concept enables the identification of potential biologically associated genes and variants for traits or diseases by studying genetic variation in the context of gene expression profiles and/or other high-throughput molecular measurements (methylome, proteome and metabolome) [30].

In the current study, systems genomics was applied by identifying expression quantitative trait loci (eQTLs) [31, 32] in liver and testis. The tissues were selected based on their biological roles within the boar taint condition: the liver is responsible for the breakdown of both skatole and androstenone, and the testis is the primary synthesis site for androstenone and other odour-causing androgens [4]. The analysis of eQTLs enables identification of *cis*-acting and *trans*-acting elements, e.g., SNPs located within gene promoters and/or distant transcription factors [33]. From the identified eQTLs, manageable lists of positional candidate SNPs and genes can be created for further investigation as potential biomarkers [34]. The strategy has been applied for traits such as hypertension [35] and obesity [33, 36] in humans and meat quality traits in pigs [37, 38]. A disadvantage of eQTL analysis is the computation time, which can amount to weeks or months because of the enormous data calculations required. Recently, an ultra-fast software package that employs large matrix operations was described for rapid eQTL identification [39]. Furthermore, the authors published a multi-tissue eQTL identification package that utilises a Bayesian hierarchical model to find eQTLs that are shared between tissues [40] and thus are highly applicable as candidate biomarkers for a given trait.

The aims of this study were to i) identify and analyse single- and multi-tissue eQTLs significantly associated with estimated breeding values (EBVs) of skatole and human nose scores (indicators of boar taint) in Danish Landrace pigs, ii) functionally characterise the SNPs and genes of eQTLs and iii) find candidate eQTLs associated with low-EBV genotypes and evaluate any possible selection effects by comparison of genomic loci with those of important production traits.

## Materials and methods

A complete illustration of the study design is available in S1 File..

### Animals and data

The animals in this study were obtained from a previously published study by our group [41]. Briefly, commercial Danish Landrace male pigs (n = 114) were produced from sires with known genetic merit of boar taint, assessed by estimated breeding values (EBVs) of skatole concentrations and human nose scores (HNS). The EBVs of the sires were obtained from the Danish pig breeding database (Pig Research Centre, SEGES, Copenhagen, Denmark). The sire EBVs were corrected for age and weight by a previously published methodology [19] to account for variances in sexual maturation of the boars and to obtain full steroidogenic potential. The pigs were produced and housed at the testing station “Bøgildgård” in Denmark (Mallingvej 1, 8620 Kjellerup, Denmark, CVR license 12739664) operated by the Pig Research Center, SEGES (Copenhagen, Denmark) with *ad libitum* feed and water supply. The authors of this study were not responsible for animal husbandry, diet and care as the testing station is a commercial facility. Furthermore, Animal Care and Use Committee accreditation was not obtained for this study because the animals were commercial slaughter pigs and tissue samples were obtained from the commercial slaughter facility “Danish Crown Pork A/S” (Danmarksgade 22, 7400 Herning, Denmark, CVR license 26121264).

Before slaughter, the pigs were grouped into one of three groups: high, medium and low genetic merit of boar taint. The grouping was performed according to the summarised EBVs of skatole and HNS obtained from the sire of each pig. The low and high groups comprised the lowest and highest sire summarised EBVs, whereas the medium group comprised pigs with sire summarised EBVs closest to the overall mean. Finally, a total of 48 pigs were selected for analysis, with 16 pigs in each of the three groups. From the selected pigs, the mean summarised EBVs were 0.71 (± 0.19), -0.01 (± 0.09) and -0.38 (± 0.17) for high, medium and low genetic merit of boar taint groups, respectively. Full information on the animals and their corresponding summarised EBVs of the sires are accessible as supplementary information in the previously published study by our group [41].

### Tissue collection and preparation

Pigs were slaughtered at a weight of ~100 kg at a commercial slaughterhouse (Danish Crown, Herning, Denmark, CVR 26121264). Slaughter was performed by submersion into CO2 until unconsciousness ensued followed by exsanguination. Following slaughter, liver and ham muscle was extracted from the carcass and 150 mg of tissue were retrieved by punch biopsy and immediately immersed into 1.5 ml RNAlater (QIAGEN, Hilden, Germany) in 2 ml Eppendorf tubes (Eppendorf, Hamburg, Germany). The carcasses were kept in a cold room at 4°C for approximately 1.5 h before 150 mg testis tissue were retrieved by punch biopsy and immersed into 1.5 ml RNAlater (QIAGEN). All samples (n = 96) were stored at -20°C for 14 days until RNA extraction and sequencing at a commercial facility (AROS A/S, Aarhus, Denmark).

### RNA extraction and sequencing

RNA extraction, sequencing and gene counting was performed as described in a previous study from our group [41]. Briefly, total RNA was extracted from the 96 samples by RNeasy Mini Kit (QIAGEN) following instructions of the manufacturer. Concentration of RNA was measured by Nanodrop 2000 (ThermoScientific, Massachusetts, USA) and quality was evaluated with a Bioanalyzer (Agilent, California, USA). Sequencing libraries were prepared from 400 ng RNA using either the TruSeq stranded mRNA (Illumina, San Diego, USA) kit following manufacturer’s instructions or the TruSeq total stranded RNA (Illumina) kit following manufacturer’s instructions according to RNA quality. Libraries were subjected to quality control with respect to size profile by test on an Agilent DNA 1000 (Agilent, California, USA) and library concentration by KAPA quantitative PCR (qPCR) kit and three independent 10^6^-fold dilutions of libraries following instructions of the manufacturer (Kapa Biosystems, Massachusetts, USA). The samples were sequenced with an Illumina HiSeq 2500 (Illumina, San Diego, USA) which amounted to a theoretical 40 million reads per sample and demultiplexed into FastQ-files by CASAVA-software (Illumina, San Diego, USA).

### Quality control and gene counting

Quality control (QC) of RNA-Seq reads was conducted with FastQC (v. 0.11.3) [42]. Reads were trimmed for known Illumina TruSeq adapter sequences using the software CutAdapt (v. 1.8.1) [43]. Poor reads were trimmed by the software Trimmomatic (v. 0.33) [44] using default parameters. The trimmed reads were then mapped to the *Sus scrofa* reference genome (10.2, version 79) by the STAR aligner (v. 020201) [45] using default parameters. Post-mapping QC was performed with Qualimap [46]. The mapped reads were counted to each gene by HTSeq count (v. 0.6.0) [47] using default parameters. All subsequent statistical analysis were performed in R (v. 3.1.0) [48]. Only genes with a mean count of more than five were included in the gene count matrices. Normalisation of gene counts was performed by voom variance-stabilization function with sample quality weights [49] implemented in the R package limma (v. 3.30.3) [50].

### Genotyping and filtering

Genomic DNA was extracted from ham muscle by DNA Minikit (QIAGEN) following manufactures instructions and genotyped using the Illumina PorcineSNP60 Beadchip (San Diego, CA, USA) [51]. Variants were called using Illumina GenomeStudio (v. 2011.1) using the Genotyping module (v. 1.9.4). To export the data into a PLINK-readable format, the PLINK Input Report plugin (v. 2.1.3) by Illumina, Inc. was used. For filtering of genotype data, PLINK [52] (v. 1.90b3.44) was used. The variants were included in the genotype data when they achieved a call rate above 0.95, a minor allele frequency (MAF) of above 0.05 and were in Hardy-Weinberg equilibrium (*P* > 0.0001). To remove variants which were in linkage disequilibrium (LD), LD-based variant pruning was performed with a window size and step size of 5 Kb and an r^2^ threshold of 0.8. Finally, the variants were converted to their genomic coordinates and Reference SNP cluster ID (rs) accession numbers which was obtained from SNPchiMp v. 3 database [53].

### Analysis of single- and multi-tissue eQTLs

#### Identification of single- and multi-tissue eQTLs

Identification of *cis-* and *trans-*acting single-tissue eQTLs was performed by the software Matrix eQTL (v. 2.1.1) [39]. The maximum distance at which a gene-SNP pair was considered a local *cis*-acting eQTL was defined as 1 Mb which was consistent with threshold used in previous research on eQTLs for obesity in pigs [33]. *P*-value thresholds were 1 × 10^−3^ for *cis*-acting eQTLs and 1 × 10^−6^ for *trans*-acting eQTLs. In accordance with guidelines for Matrix eQTL, different thresholds for each eQTL type was applied [39]. This was due to a limitation in the Matrix eQTL software as it was not possible to apply FDR thresholds. Hence, diverging *P* value thresholds were set to avoid excessive large output files as only a small amount of the identified eQTLs passed the FDR threshold (FDR < 0.05). The grouping of the pigs as low, medium or high boar taint by their summarised EBVs of skatole and HNS was loaded into the software as covariates in the model.

Identification of multiple tissue (multi-tissue) eQTLs which comprised eQTLs identified in both liver and testis was performed using the Matrix eQTL extension named Empirical Bayesian Hierarchical Model [40]. Subsequently, all eQTLs were ordered according to their Benjamini-Hochberg adjusted *P*-values (FDR). An association analysis was performed on both single- and multi-tissue eQTLs to test for association with: i) estimated breeding values (EBVs) for skatole and human nose score (HNS) of the animals and ii) the expression profile of the gene associated with the eQTL. This was performed by comparing the summarised EBVs and expression profiles from the animals that were naturally grouped by one of the three available genotypes (AA / AB / BB) in each individual eQTL by a linear regression model. To account for any batch or slaughter line-derived biological effects, tissue yield (mg), quality control data from the genotyping machine and the RNA integrity number (RIN) of each sample were included as interacting covariates in the model. Subsequently, test statistics and *P*-values were computed with ANOVA tests by the function anova() in the R package stats (v. 3.3.1) [48]. Finally, the *P* values were subjected to stringent multiple testing corrections by the FDR procedure. The eQTLs with significant (FDR < 0.05) association to summarised EBVs and gene expression were defined as the “filtered eQTLs”. The filtered *cis*-acting eQTLs from both single- and multi-tissue were used for functional characterisation and evaluation as candidate eQTLs.

#### Chromosomal statistics of eQTLs

To provide descriptive statistics on the eQTLs, chromosomal locations together with lengths of the *Sus scrofa* chromosomes, were obtained by the R package biomaRt (v. 2.30.0) [54, 55] from the *Sus scrofa* genome build (10.2, version 79). The chromosomal locations of the SNPs associated with the eQTLs were obtained from SNPchiMp (v. 3) database [53]. The chromosomal densities of eQTLs were calculated by the number of either unfiltered or filtered eQTLs per chromosome divided by length in bases of the respective chromosome.

#### Functional characterisation and pathway analysis

Functional characterisation was performed by the Cytoscape (v. 3.2.1) [56] plug-in ClueGO (v. 2.2.5) [57]. By providing the genes associated with the filtered *cis*-acting eQTLs from liver and testis, ClueGO performed an overrepresentation test which provided enriched functions and pathways in the gene subset. Furthermore, the tool visualised significantly (FDR < 0.05) enriched gene ontology (GO) terms of each gene. Finally, ClueGO visualised the expression profiles of “low” and “high” boar taint groups by a scale of red to green. Red colour represented upregulation and green colour represented downregulation, as compared to the mean expression values of all three groups. Results from enrichment test were subjected to multiple testing corrections of the *P* values by Benjamini-Hochberg (FDR) correction.

#### Correlation analysis between multi-tissue eQTLs

Correlation analysis was performed to reveal co-expression patterns of genes associated with multi-tissue eQTLs. The analysis was performed in ClueGO. Correlations were calculated Spearman rank correlation between expression levels of all multi-tissue eQTLs from both liver and testis. The correlations were visualised as a network plot in Cytoscape. Each node represented a gene associated with multi-tissue eQTLs and edges represented the results from the correlation analyses. Edges from correlations calculated from both liver and testis expression data were included and coloured identically. However, positive correlations were coloured red whereas negative correlations were coloured green, consistent with the colours used by other network co-expression analyses tools, e.g. Weighted Gene Co-expression Network Analysis (WGCNA) [58]. All positive and negative correlations with R^2^ ≥ 0.5 were visualised.

#### Genomic location analysis and QTL trait enrichment test

To find and evaluate possible selection effects, genomic coordinates from known traits represented by their quantitative trait loci (QTLs) were obtained from the Animal Genome PigQTLdb database where all previous research on QTLs are curated. The known QTL traits from the database were tested for enrichment by the number of genomic overlaps of the filtered *cis*-acting eQTLs from both liver and testis. This was done using the R package binom.test.QTL [59]. Briefly, the package tests each trait in a global QTL dataframe obtained from the PigQTLdb [22] against SNPs associated with the eQTLs. In this study, a total of 646 unique porcine traits were obtained from the PigQTLdb and used in the enrichment test, which were represented by a total of 25,473 QTLs and their corresponding genomic positions (updated December 2017). The enrichment test was performed by obtaining the number of expected overlaps between the filtered multi-tissue eQTLs and the known traits and comparing the expected overlaps by a binominal test with the full length of the porcine genome as the background set. The expected overlaps were calculated as the number of intersections between the genomic locations of the SNPs associated with the filtered multi-tissue eQTLs and the summarised length of the QTLs which were associated with each database-derived trait. The background set was defined as the full length (2.7 Gb) of the porcine genome. Finally, the *P*-values were subjected to multiple testing corrections by the Benjamini-Hochberg (FDR) procedure. In order to obtain overlaps from the most relevant breeds and filter out esoteric and local breeds, only QTLs represented by breeds with a minimum of a thousand QTLs were included in the analysis. After filtering, a total of 577 traits represented by 19,417 QTLs were included in the final analysis. The most represented breeds were Duroc × Erhulian, Duroc × Pietrain and Duroc × Yorkshire/Landrace. The package also provided the category of each of the enriched trait. To adjust for any bias due to higher research interests in particular production traits, the sum of enriched traits from each category were divided with the sum of all traits from the corresponding category in the current Animal Genome PigQTLdb (release 32).

#### Selection of candidate eQTLs

In order to select the most interesting subset of eQTLs with potential usefulness in the development of candidate biomarkers for gene-based selection schemes towards lowered boar taint, a list of candidate eQTLs was generated from the filtered *cis*-acting eQTLs that fulfilled the two criteria: i) associated with genotype(s) with a median of summarised EBVs equal to or lower than the maximum summarised EBVs of the low boar taint pig group (low group EBV_max_ = -0.070) and ii) significantly (FDR < 0.05) overlapping a known boar taint relevant QTL trait by its genomic coordinates. Finally, the candidate eQTLs were visualised plotted with expression levels from liver and testis and its candidate genotype associated with low summarised EBVs for skatole and HNS.

## Results

### Quality control and filtering

#### RNA-Seq data

All RNA-Seq data was obtained from a previous study on differentially expressed genes (DEG) associated with the summarised estimated breeding values (EBVs) of skatole and human nose scores (HNS) [41]. Briefly, post-mapping quality control revealed a mean of 33.25 million reads successfully mapped to the *Sus scrofa* Ensembl reference genome (10.2, version 79). Furthermore, reads aligned to genes (± SD) were 7.76 (± 1.61) million and 7.04 million (± 2.44) in liver and testis, respectively. To remove genes with none or very low expression levels, only genes with mean expression levels of more than five counts were included in the analysis. This filtering approach was consistent with previous eQTL analysis in the porcine genome [33] and consistent with previous warrants to obtain the highest statistical power [60]. The final numbers of genes were 10,545 for liver and 12,731 for testis, respectively.

#### Genotype data

Genotype data comprised a total of 59,319 SNPs with a total genotyping rate of 0.997751. Due to missing genotype data, 567 variants removed. Furthermore, 68 SNPs were removed due to Hardy-Weinberg exact test filtering and 19,843 SNPs were removed due to minor allele threshold. Linkage disequilibrium (LD) based variant pruning removed 13,186 variants due to high LD (r^2^ ≥ 0.8) and a final subset of 20,827 SNPs was used for the analysis.

### Analysis of single-tissue eQTLs

#### Identification of single-tissue eQTLs in liver

**Fig 1:**
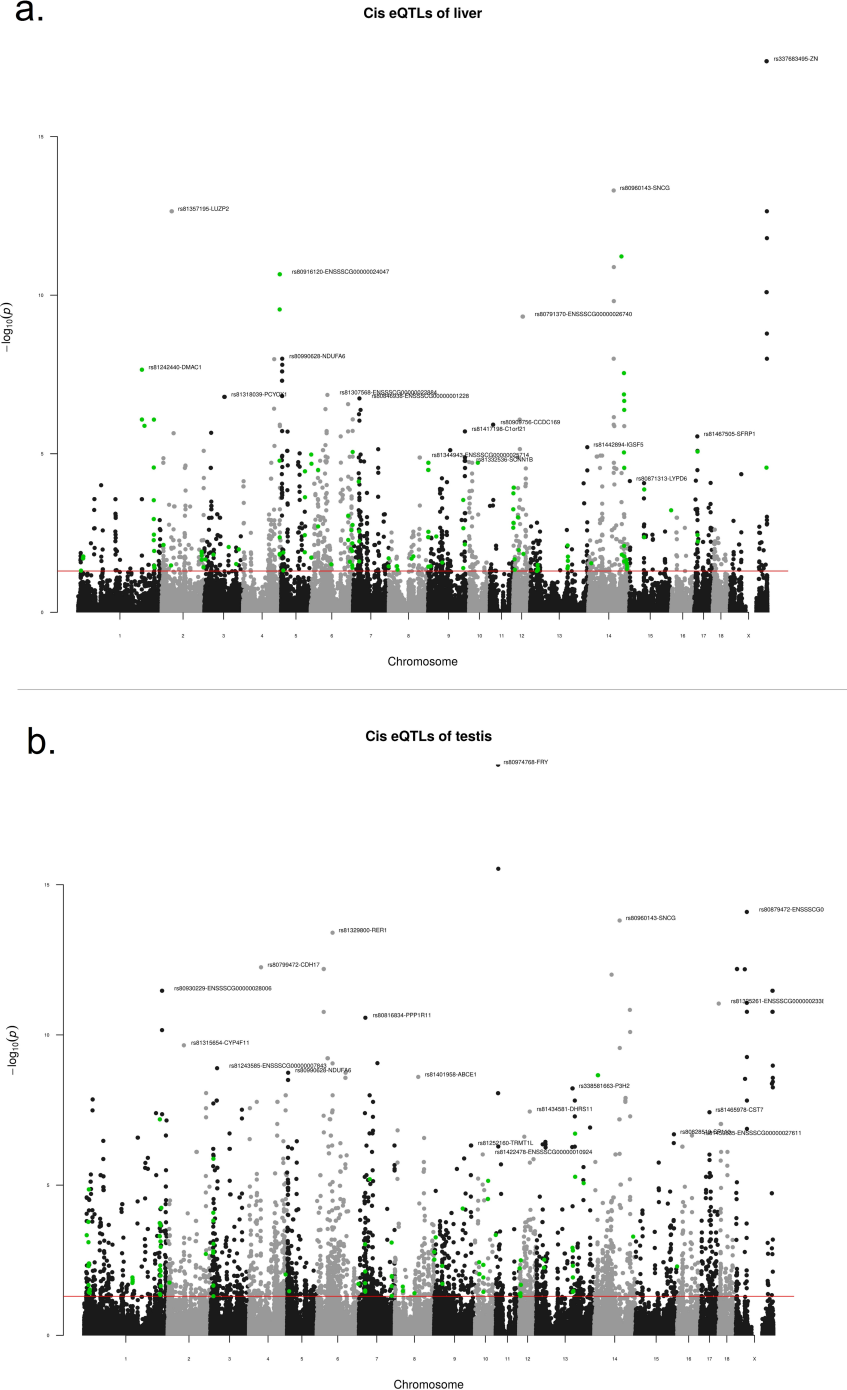
**Identification of significant *cis*-acting eQTLs in porcine (a) liver and (b) testis.** Y-axis indicates the negative logarithm of False Discovery Rate for each eQTL, X-axis indicates chromosome number. Only the X chromosome is shown, as no eQTLs were discovered on Y chromosome. Red line indicates a FDR threshold of 0.05 which defined the eQTLs with a significant gene-SNP association. For each chromosome, the eQTLs with lowest FDR values are illustrated with SNP rs number and gene symbol. Green circles indicate “filtered eQTLs” which had a significant (FDR < 0.05) association with sire-obtained summarised estimated breeding values for skatole and human nose scores and were used in the subsequent analyses.

A total of 1,949 *cis*-acting and 851 *trans*-acting expression quantitative trait loci (eQTLs) with significant (FDR < 0.05) gene-SNP relationship were identified in liver (Fig 1A). Association analysis revealed 142 *cis*- and 63 *trans*-acting eQTLs with significant association (FDR < 0.05) to the summarised EBVs of skatole and HNS. These eQTLs were defined as the “filtered eQTLs” of liver and selected for functional analysis and QTL trait enrichment analysis for candidate eQTLs. The *cis*- and *trans*-acting filtered eQTLs were associated with 85 and 17 genes, respectively. The top 30 *cis*-acting eQTLs were associated with the genes *ARMC7*, *UROS*, *ZNF662*, *BAIAP2*, *FAM198A*, *ENSSSCG00000024047*, *TEN1*, *ENSSSCG00000020732*, *ENSSSCG00000005474*, *NEB*, *HEXDC*, *ATP13A3*, *LSG1*, *BUB3*, *ACADSB* and *GSTA4* (Table 1). Full results table are available in S2 File.

**Table 1:** Top 30 *cis*-acting filtered eQTLs in porcine liver.

SNP	Gene	FDR-eQTL	FDR- EBV	SNP- Chr	Distance (bp)	GO term
rs81265837	*ARMC7*	0.045	0.000	12	-871	molecular function
rs81435284	*ARMC7*	0.045	0.000	12	-92982	molecular function
rs80892090	*UROS*	0.038	0.002	14	702737	biological process
rs80822699	*ZNF662*	0.044	0.002	13	-48202	organelle
rs81444147	*ZNF662*	0.044	0.002	13	-205961	organelle
rs81347708	*ZNF662*	0.044	0.002	13	-411021	organelle
rs81444056	*ZNF662*	0.047	0.002	13	160293	organelle
rs81305046	*BAIAP2*	0.000	0.002	12	-111914	cell morphogenesis
rs81438833	*BAIAP2*	0.000	0.002	12	-404940	cell morphogenesis
rs81439307	*BAIAP2*	0.000	0.002	12	-552982	cell morphogenesis
rs81440749	*BAIAP2*	0.000	0.002	12	-1009083	cell morphogenesis
rs81268762	*FAM198A*	0.032	0.003	13	979078	organelle
rs80871264	*ENSSSCG00000024047*	0.004	0.006	4	348015	
rs81433762	*TEN1*	0.021	0.006	12	238431	biosynthetic process
rs81434107	*TEN1*	0.021	0.006	12	183314	biosynthetic process
rs80878429	*ENSSSCG00000020732*	0.004	0.007	5	634366	
rs81444193	*ZNF662*	0.042	0.008	13	-800329	organelle
rs81351832	*ENSSSCG00000005474*	0.000	0.009	1	214191	
rs80905121	*ZNF662*	0.040	0.011	13	-6078	organelle
rs81456636	*NEB*	0.001	0.013	15	27586	extracellular region
rs81311681	*HEXDC*	0.001	0.013	12	-10300	molecular function
rs81434341	*HEXDC*	0.001	0.013	12	-315178	molecular function
rs81271924	*HEXDC*	0.001	0.013	12	-394460	molecular function
rs81286188	*ATP13A3*	0.024	0.014	13	-753004	biological process
rs81286188	*LSG1*	0.036	0.014	13	-469274	biological process
rs80871231	*BUB3*	0.032	0.015	14	-853556	molecular function
rs80871231	*ACADSB*	0.043	0.015	14	-967074	molecular function
rs80799070	*ENSSSCG00000005474*	0.004	0.016	1	255068	
rs81257157	*ENSSSCG00000020732*	0.013	0.016	5	768093	
rs80957762	*GSTA4*	0.020	0.016	7	1429	molecular function

SNP = Single nucleotide polymorphism, FDR-eQTL = False Discovery Rate from association test between gene and SNP in the eQTL calculated by Matrix eQTL software, FDR-EBV = False Discovery Rate from association test between eQTL and summarised estimated breeding values (EBVs) of skatole and human nose scores, SNP-Chr = Chromosome of the SNP associated with the eQTL, Distance = Genomic distance between gene-SNP pair in bases. Negative number indicates upstream location of SNP respective to gene, GO Term = Gene Ontology term associated with the eQTL.

#### Identification of single-tissue eQTLs in testis

A total of 4,442 *cis*-acting and 1,437 *trans*-acting eQTLs with significant (FDR < 0.05) gene-SNP relationship were identified in testis (Fig 1B). Association analysis revealed 77 *cis*- and 32 *trans*-acting eQTLs with significant association (FDR < 0.05) to the summarised EBVs of skatole and HNS. These eQTLs were defined as the “filtered eQTLs” of testis and selected for functional analysis and QTL trait enrichment analysis for candidate eQTLs. The *cis*- and *trans*-acting filtered eQTLs were associated with 47 and 11 genes, respectively. The top 30 *cis*-acting eQTLs were associated with the genes *GTF2I*, *ENSSSCG00000025739*, *NPTX1*, *TEPSIN*, *CCDC170*, *SERPINA3*, *NWD2*, *SNX30*, *ESR1*, *ENSSSCG00000028881* and *FBP2* (Table 2). Full results table are available in S2 File.

**Table 2:** Top 30 *cis*-acting filtered eQTLs in porcine testis.

SNP	Gene	FDR-eQTL	FDR- EBV	SNP- Chr	Distance (bp)	GO term
rs81265837	*ARMC7*	0.003	0.000	12	-871	molecular function
rs81435284	*ARMC7*	0.003	0.000	12	-92982	molecular function
rs81286957	*GTF2I*	0.002	0.003	3	432935	cytoplasm
rs81371954	*GTF2I*	0.002	0.006	3	343502	cytoplasm
rs81223031	*GTF2I*	0.050	0.006	3	-299269	cytoplasm
rs80892090	*UROS*	0.001	0.007	14	702737	biological process
rs81444403	*ENSSSCG00000025739*	0.003	0.008	13	-387191	
rs81305046	*NPTX1*	0.006	0.008	12	285025	cell-cell signalling
rs81438833	*NPTX1*	0.006	0.008	12	-8001	cell-cell signalling
rs81439307	*NPTX1*	0.006	0.008	12	-156043	cell-cell signalling
rs81440749	*NPTX1*	0.006	0.008	12	-612144	cell-cell signalling
rs81435983	*ARMC7*	0.021	0.008	12	-348155	molecular function
rs81321757	*ARMC7*	0.021	0.008	12	-491525	molecular function
rs81305046	*TEPSIN*	0.042	0.008	12	-222102	molecular function
rs81438833	*TEPSIN*	0.042	0.008	12	-515128	molecular function
rs81439307	*TEPSIN*	0.042	0.008	12	-663170	molecular function
rs80814798	*CCDC170*	0.005	0.009	1	896311	molecular function
rs81348764	*CCDC170*	0.005	0.009	1	-25868	molecular function
rs80871264	*ENSSSCG00000024047*	0.009	0.009	4	348015	
rs81348677	*CCDC170*	0.021	0.009	1	499103	molecular function
rs81328558	*CCDC170*	0.021	0.009	1	349781	molecular function
rs80982470	*SERPINA3*	0.001	0.020	7	-228084	lysosome
rs81398873	*NWD2*	0.024	0.020	8	-114091	
rs80993397	*CCDC170*	0.036	0.020	1	-263557	molecular function
rs81351832	*SNX30*	0.000	0.022	1	61251	cell
rs80836463	*ESR1*	0.000	0.022	1	686066	signal transducer activity
rs81348579	*ESR1*	0.000	0.022	1	566976	signal transducer activity
rs81348614	*ESR1*	0.001	0.022	1	261590	signal transducer activity
rs81420611	*ENSSSCG00000028881*	0.004	0.022	10	-88026	
rs81288871	*FBP2*	0.005	0.022	10	1026726	ion binding

SNP = Single nucleotide polymorphism, FDR-eQTL = False Discovery Rate from association test between gene and SNP in the eQTL calculated by Matrix eQTL software, FDR-EBV = False Discovery Rate from association test between eQTL and summarised estimated breeding values (EBVs) of skatole and human nose scores, SNP-Chr = Chromosome of the SNP associated with the eQTL, Distance = Genomic distance between gene-SNP pair in bases. Negative number indicates upstream location of SNP respective to gene, GO Term = Gene Ontology term associated with the eQTL.

#### Chromosomal statistics of single-tissue eQTLs

Pig chromosomes have the following densities of all eQTLs, ordered from highest to lowest: SSC6 (8.06), SSC14 (7.89), SSC12 (4.97), SSC4 (4.87), SSC7 (4.08), SSC5 (3.87), SSC3 (3.35), SSC17 (3.34), SSC2 (2.87), SSC9 (2.79), SSC18 (2.52), SSC1 (2.29), SSC13 (2.12), SSC8 (2.05), SSC10 (1.83), SSC16 (1.83), SSC11 (1.37), SSCX (1.24), SSC15 (1.17) and SSCY (0). Pig chromosomes have the following densities of filtered *cis*- and *trans*-acting eQTLs, ordered from highest to lowest: SSC12 (0.519), SSC1 (0.415), SSC13 (0.338), SSC9 (0.299), SSC14 (0.279), SSC10 (0.265), SSC2 (0.215), SSC3 (0.173), SSC7 (0.171), SSC6 (0.165), SSC4 (0.146), SSC5 (0.135), SSC17 (0.0717), SSC15 (0.0571), SSC8 (0.0539), SSCX (0.0139), SSC11 (0.0114), SSC18 (0), SSC16 (0) and SSCY (0).

#### Functional characterisation and pathway analysis of the single-tissue eQTLs

**Fig 2:**
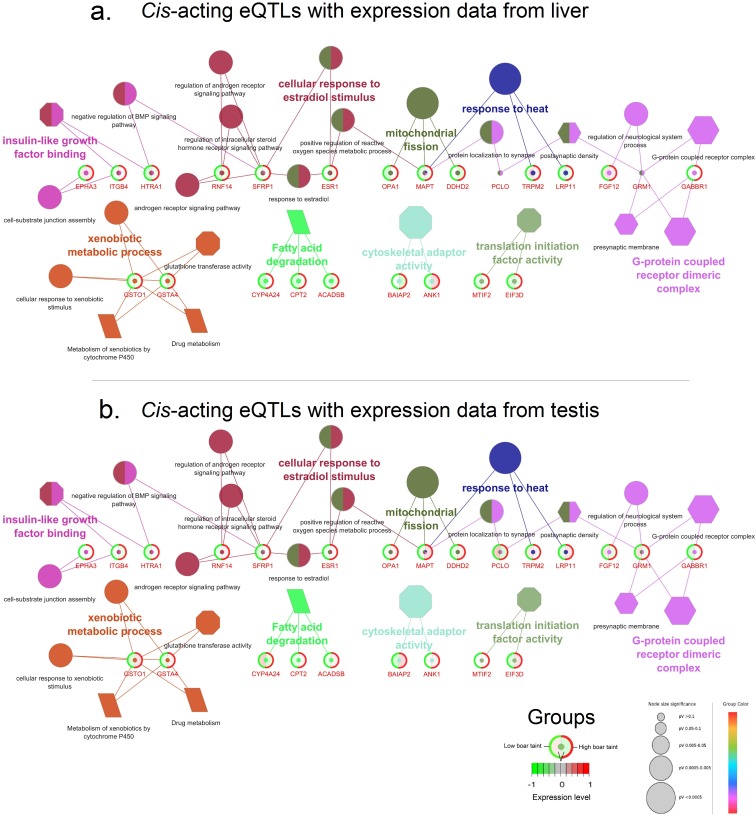
Functional characterisation and pathway analysis of the filtered *cis*-acting eQTLs. Filtered *cis*-acting eQTLs with expression data (a) liver and (b) testis were subjected to functional characterisation and pathway analysis. Functional characterisation is represented by gene ontology (GO) terms and pathways are represented by their Kyoto Encyclopedia Genes and Genomes (KEGG) pathways. Each function/pathway is visualised by a uniquely coloured symbol. Each gene is represented by a circle where the colour in the centre indicates the function of the gene from the corresponding GO term. The diameter of the circle is negatively correlated with the adjusted *P* value (FDR) of the GO term from the functional enrichment test made by ClueGO software. Each node represents a gene where the expression levels are indicated in the circle where the green group (left side) represents the expression level in low boar taint pigs and the red group (right side) represents the expression level in high boar taint pigs. *Ellipse symbol*: Biological process; *hexagon symbol*: cellular component; *octagon symbol*: molecular function; *parallelogram*: KEGG pathway.

Functional characterisation revealed 31 and three GO terms and KEGG pathways to be significantly (FDR < 0.05) enriched from the filtered single-tissue *cis*-acting eQTLs, respectively (Fig 2). Of these, biological relevant GO terms sorted according to FDR included “Xenobiotic metabolic process”, “Cellular response to xenobiotic stimulus”, “Cellular response to estradiol stimulus”, “Regulation of androgen receptor signalling pathway”, “Glutathione transferase activity”, “Response to estradiol” “Androgen receptor signalling pathway” and “Regulation of intracellular steroid hormone receptor signaling pathway” (Fig 2). Pathway analysis revealed three KEGG pathways to be significantly (FDR < 0.05) enriched: “Metabolism of xenobiotics by cytochrome P450”, “Drug metabolism” and “Fatty acid degradation”. Full results table are available in S3 File.

### Multi-tissue eQTL analysis

#### Identification of multi-tissue eQTLs from liver and testis

By a Bayesian Hierarchical Model approach implemented in Matrix eQTL software, eQTLs shared between liver and testis was identified. These were defined as “multi-tissue eQTLs”. A total of *cis*-acting multi-tissue eQTLs were identified as significant (FDR < 0.05) by their gene-SNP relationship. Association analysis revealed a total of 26 eQTLs as significantly associated (FDR < 0.05) with summarised EBVs for skatole and HNS. These were defined as the “filtered multi-tissue eQTLs” and were associated with the genes *ARMC7*, *UROS*, *ENSSSCG00000024047*, *NEB*, *ENSSSCG00000029075*, *TMEM41B*, *SYTL3*, *ZNF185*, *DMAC1*, *ENSSSCG00000025800*, *KLHDC4* and *CES1* (Table 3). Full results table are available in S4 File.

**Table 3:** Filtered multi-tissue eQTLs from liver and testis.

SNP	Gene	FDR-expression	FDR- EBV	SNP Chr	Distance (bp)	GO term
rs81265837	*ARMC7*	0.005	0.000	12	-871	molecular function
rs81435284	*ARMC7*	0.005	0.000	12	-92982	molecular function
rs80892090	*UROS*	0.038	0.001	14	702737	biological process
rs80871264	*ENSSSCG00000024047*	0.000	0.003	4	348015	
rs81456636	*NEB*	0.000	0.008	15	27586	extracellular region
rs80947338	*ENSSSCG00000029075*	0.000	0.021	14	-673630	
rs81411161	*TMEM41B*	0.000	0.021	9	-299322	biological process
rs81313669	*TMEM41B*	0.000	0.021	9	-435492	biological process
rs339471111	*TMEM41B*	0.000	0.021	9	-681330	biological process
rs81270995	*TMEM41B*	0.000	0.021	9	231435	biological process
rs81411123	*TMEM41B*	0.000	0.021	9	-51275	biological process
rs81477176	*TMEM41B*	0.000	0.021	9	-721167	biological process
rs80932898	*SYTL3*	0.035	0.021	1	512807	molecular function
rs81479582	*ZNF185*	0.000	0.022	X	965559	molecular function
rs80916120	*ENSSSCG00000024047*	0.000	0.022	4	382514	
rs80919384	*ENSSSCG00000024047*	0.000	0.022	4	128566	
rs337275357	*ENSSSCG00000024047*	0.000	0.022	4	-140288	
rs81242440	*DMAC1*	0.000	0.022	1	503055	cytoplasm
rs80922101	*DMAC1*	0.000	0.022	1	311763	cytoplasm
rs80830287	*ENSSSCG00000025800*	0.001	0.022	14	-440700	
rs80948915	*ENSSSCG00000024047*	0.000	0.022	4	550515	
rs80906401	*ENSSSCG00000025800*	0.000	0.023	14	201338	
rs81323702	*KLHDC4*	0.000	0.026	6	89447	molecular function
rs81380844	*ENSSSCG00000024047*	0.000	0.041	4	982688	
rs81320759	*KLHDC4*	0.000	0.041	6	-812563	molecular function
rs81395038	*CES1*	0.000	0.041	6	-150074	intracellular

SNP = Single nucleotide polymorphism, FDR-expression = False Discovery Rate from association test between eQTL and expression profile of the associated gene, FDR-EBV = False Discovery Rate from association test between eQTL and summarised estimated breeding values (EBVs) of skatole and human nose scores, SNP Chr = Chromosome of the SNP associated with the eQTL, Distance = Genomic distance between gene and SNP pair in the eQTL in bases. Negative number indicates upstream location of SNP respective to gene, GO Term = Gene Ontology of the gene associated with the eQTL.

#### Correlation analysis between expression levels of multi-tissue eQTLs

**Fig 3:**
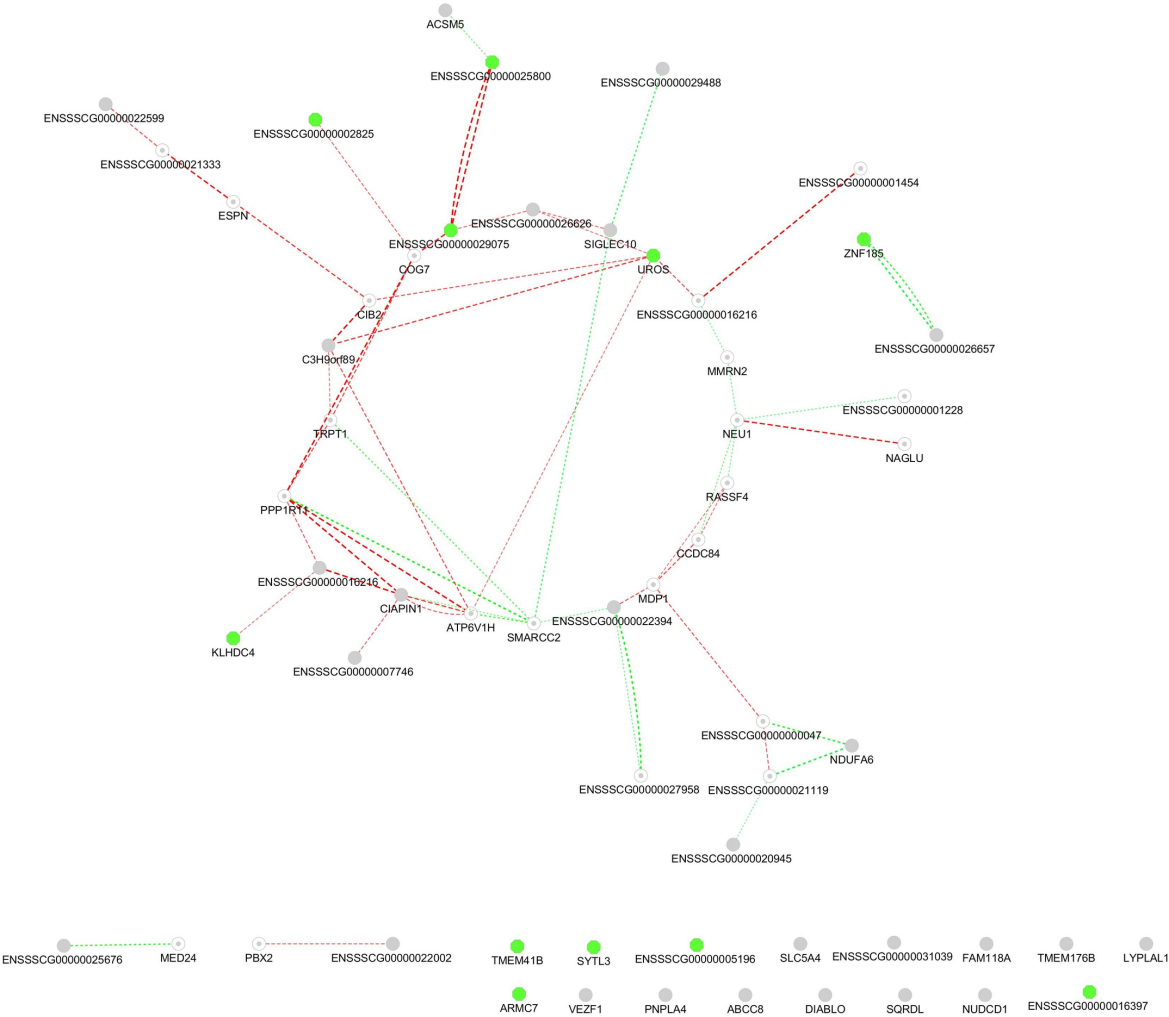
Correlations analysis between expression levels of multi-tissue eQTLs. To identify co-expression patterns between multi-tissue eQTLs, correlations of expression levels of genes associated with multi-tissue eQTLs was analysed by Spearman rank correlation analysis. Correlations were calculated from expression data from both liver and testis. Edges indicating correlation are shown for both liver and testis expression data. Green circles indicate filtered multi-tissue eQTLs with significant association to summarised EBVs for skatole and HNS. The two types of correlations are discerned by both colour and shape. Positive correlations between expression levels are indicated with dashed red line. Negative correlations between expression levels are indicated by a dashed green line. Boldness of lines indicates the degree of correlation. Only edges representing Spearman rank correlations above R^2^ > 0.5 or below R^2^ < -0.5 are shown.

To identify multi-tissue eQTLs that were co-expressed with other multi-tissue eQTLs, a correlation analysis by Spearman rank correlation analysis was performed on all multi-tissue eQTLs identified with significant gene-SNP relationship which included the filtered multi-tissue eQTLs. The filtered multi-tissue eQTLs were indicated with red letters in the illustration. Correlation analysis revealed multi-tissue eQTLs associated with the genes *PPP1R11*, *NEU1*, *SMARCC2*, *UROS* and *CIAPIN1* to contain most correlations to and from other multi-tissue eQTLs (threshold: R^2^ ≥ 0.5) (Fig 3).

#### Genomic location analysis and QTL trait enrichment test

Enrichment test for selection effects revealed 89 QTL traits from Animal Genome PigQTLdb to be significantly (FDR < 0.05) enriched by genomic overlaps from a total of 206 filtered *cis*-acting eQTLs from liver and testis. The top five enriched traits were “Palmitic acid to myristic acid ratio”, “Vertebra number”, “Loin muscle area”, “Ear area” and “Ear weight” (Table 4).

**Table 4:** Top 30 enriched QTL traits from the filtered *cis*-acting eQTLs of liver and testis.

Known QTL trait	Genes from eQTLs with significant overlap	FDR
Palmitic acid to myristic acid ratio	*ENSSSCG00000020784*, *LATS1*	5.816E-16
Vertebra number	*ENSSSCG00000029150*, *CCDC170*, *SNX30*, *ESR1*, *ZWILCH*, *TIPIN*, *ENSSSCG00000004117*, *GRM1*, *ENSSSCG00000005474*, *TNC*, *ENSSSCG00000020784*, *SYTL3*, *LATS1*, *ASTN2*, *LRP11*, *GOLM1*, *ENSSSCG00000028881*, *FBP2*, *CTSL*, *ARMC7*, *BAIAP2*, *TEN1*, *HEXDC*, *INTS2*, *AOC2*, *ATP13A3*, *ENSSSCG00000029252*, *ENSSSCG00000011817*, *KALRN*, *OPA1*, *ACADSB*, *ENSSSCG00000029075*, *ENSSSCG00000025800*, *ENSSSCG00000010634*, *ENSSSCG00000009663*, *UROS*, *EXTL3*	1.009E-15
Loin muscle area	*ENSSSCG00000005474*, *ENSSSCG00000022796*, *TMEM242*, *CCDC170*, *SNX30*, *ESR1*, *ZWILCH*, *TIPIN*, *ENSSSCG00000004117*, *GRM1*, *TNC*, *ENSSSCG00000020784*, *CTSL*, *TEN1*, *HEXDC*, *MAPT*, *BAIAP2*, *ARMC7*, *NPTX1*, *TEPSIN*, *ITGB4*, *ZNF662*, *ATP13A3*, *LSG1*, *PLCD1*, *EPHA3*, *KALRN*, *UROS*, *BUB3*, *EXTL3*, *NEB*, *DDHD2*, *MAP3K20*, *ANK1*, *BBOX1*, *NAALADL1*, *FAM49A*, *FNDC4*, *MTIF2*, *AP5Z1*, *CARHSP1*, *ENSSSCG00000024047*	5.074E-09
Ear area	*FBP2*, *NAALADL1*, *CARHSP1*, *ENSSSCG00000022090*	1.359E-08
Ear weight	*ENSSSCG00000029252*, *ENSSSCG00000011817*, *KALRN*, *OPA1*, *NAALADL1*, *ENSSSCG00000022090*	1.946E-06
PH for Biceps femoris	*CCDC170*, *SNX30*, *ESR1*, *ZWILCH*, *TIPIN*, *ENSSSCG00000004117*, *GRM1*, *ENSSSCG00000005474*, *TNC*, *ENSSSCG00000020784*	2.762E-06
pH for Semispinalis Dorsi	*CCDC170*, *SNX30*, *ESR1*, *ZWILCH*, *TIPIN*, *ENSSSCG00000004117*, *GRM1*, *ENSSSCG00000005474*, *TNC*, *ENSSSCG00000020784*	2.762E-06
Average backfat thickness	*ENSSSCG00000005474*, *NUP43*, *TNC*, *ENSSSCG00000026492*, *DMAC1*, *ENSSSCG00000022796*, *TMEM242*, *ENSSSCG00000029150*, *CCDC170*, *SNX30*, *ESR1*, *ZWILCH*, *TIPIN*, *ENSSSCG00000004117*, *GRM1*, *ENSSSCG00000020784*, *FBP2*, *CTSL*, *ARMC7*, *MAPT*, *BAIAP2*, *TEPSIN*, *ITGB4*, *ZNF662*, *ATP13A3*, *LSG1*, *PLCD1*, *ENSSSCG00000029252*, *ENSSSCG00000011817*, *ENSSSCG00000025800*, *ENSSSCG00000029075*, *ENSSSCG00000010634*, *HTRA1*, *GSTO1*, *EXTL3*, *NEB*, *BBOX1*, *NAALADL1*, *FNDC4*, *ENSSSCG00000024047*, *ENSSSCG00000022090*	5.032E-06
Thoracic vertebra number	*CCDC170*, *SNX30*, *ESR1*, *ZWILCH*, *TIPIN*, *ENSSSCG00000004117*, *GRM1*, *ENSSSCG00000005474*, *TNC*, *ENSSSCG00000020784*, *LATS1*, *ASTN2*, *LRP11*, *GOLM1*, *ENSSSCG00000028881*, *FBP2*, *CTSL*, *EXTL3*, *NEB*, *MAP3K20*, *ENSSSCG00000022090*	7.896E-06
External fat on ham	*ENSSSCG00000022796*, *TMEM242*, *ARMC7*, *ZNF662*, *ATP13A3*, *LSG1*, *ENSSSCG00000029252*, *ENSSSCG00000011817*, *NEB*, *ENSSSCG00000024047*	8.209E-06
Shoulder external fat weight	*ENSSSCG00000022796*, *TMEM242*, *ENSSSCG00000029150*, *CCDC170*, *PLCD1*, *ENSSSCG00000029252*, *ENSSSCG00000011817*, *NEB*, *NAALADL1*, *ENSSSCG00000024047*	8.209E-06
Myristic acid content	*LATS1*, *ASTN2*, *SNX30*, *LRP11*, *ENSSSCG00000020784*, *TNC*, *GOLM1*, *ENSSSCG00000028881*, *FBP2*	1.773E-05
Feed intake	*ENSSSCG00000022796*, *TMEM242*, *ARMC7*, *ZNF662*, *ATP13A3*, *LSG1*, *PLCD1*, *ENSSSCG00000010634*, *GSTO1*, *ENSSSCG00000024047*	5.929E-05
cis-11-Eicosenoic acid content	*LATS1*, *ASTN2*, *SNX30*, *LRP11*, *NUDT22*	6.655E-05
Plateletcrit	*ENSSSCG00000005474*, *CARHSP1*, *GTF2I*, *RCC1L*	6.655E-05
Neck weight	*ENSSSCG00000005474*, *NUP43*, *TNC*, *ENSSSCG00000026492*, *DMAC1*, *ENSSSCG00000022796*, *ENSSSCG00000024047*	8.269E-05
Conductivity 24 hours postmortem (loin)	*ENSSSCG00000022796*, *TMEM242*, *SNX30*, *CTSL*, *TEPSIN*, *ITGB4*, *EXTL3*, *NEB*	1.006E-04
Liver weight	*ENSSSCG00000022796*, *TMEM242*, *NEB*, *BBOX1*, *NAALADL1*, *FNDC4*, *CLDN4*, *RCC1L*, *ENSSSCG00000024047*	1.225E-04
PRRSV antibody titer	*ENSSSCG00000022796*, *TMEM242*	1.272E-04
Loin muscle depth	*ENSSSCG00000022796*, *TMEM242*, *CTSL*, *TEN1*, *HEXDC*, *NPTX1*, *ARMC7*, *TEPSIN*, *ATP13A3*, *PLCD1*, *NEB*, *BBOX1*, *NAALADL1*	1.803E-04
Muscle moisture percentage	*FBP2*, *CTSL*, *BAIAP2*, *ATP13A3*, *NEB*, *DDHD2*, *MAP3K20*, *ANK1*, *SFRP1*, *BBOX1*, *NAALADL1*, *ENSSSCG00000024047*	2.647E-04
CD8-negative leukocyte percentage	*SYTL3*, *TNC*, *ENSSSCG00000020784*, *EPHA3*, *KALRN*	2.791E-04
CD8-positive leukocyte percentage	*SYTL3*, *TNC*, *ENSSSCG00000020784*, *EPHA3*, *KALRN*	2.791E-04
**Off-Flavor Score**	***ATP13A3*, *PLCD1*, *EPHA3*, *KALRN*, *ENSSSCG00000029252*, *ENSSSCG00000011817*, *ENSSSCG00000024047***	5.498E-04
CD2-positive leukocyte number	*ENSSSCG00000022796*, *TMEM242*	6.638E-04
Inside ham weight	*FBP2*, *ENSSSCG00000029429*, *ENSSSCG00000011035*, *ARMC7*, *ENSSSCG00000025800*, *ENSSSCG00000029075*, *ENSSSCG00000010634*, *HTRA1*, *GSTO1*	7.146E-04
CD4-positive leukocyte number	*ENSSSCG00000022796*, *TMEM242*, *CARHSP1*	8.192E-04
Abdominal fat weight	*ENSSSCG00000022796*, *TMEM242*, *MAPT*, *BAIAP2*, *ZNF662*, *ATP13A3*, *LSG1*, *PLCD1*, *P3H2*, *ENSSSCG00000029252*, *ENSSSCG00000011817*, *KALRN*, *OPA1*, *NEB*, *BBOX1*, *NAALADL1*, *FAM49A*, *FNDC4*, *MTIF2*, *AP5Z1*, *CARHSP1*, *ENSSSCG00000024047*, *ENSSSCG00000022090*	9.920E-04
Days to 105 kg	*GTF2I*, *RCC1L*, *CLDN4*	9.920E-04
Linolenic acid content	*LRP11*, *ENSSSCG00000020784*, *TNC*, *GOLM1*, *ENSSSCG00000028881*, *FBP2*, *ENSSSCG00000025800*, *ENSSSCG00000029075*, *ENSSSCG00000010634*, *HTRA1*, *GSTO1*	1.164E-03

Table of known QTL traits enriched from the genomic coordinates of the filtered *cis*-acting eQTLs. Bold letters indicate traits with boar taint relevance. Known QTL trait column indicates the name of the trait from the Animal Genome PigQTLdb database. Gene column indicates genes associated with eQTLs with significant genomic overlap with known traits. FDR column indicates Benjamini-Hochberg adjusted *P*-values obtained from QTL enrichment test. Briefly, a binominal test was performed on the intersections between the genomic locations of each eQTL compared with the genomic locations of all known QTLs on a background defined as the full length (2.7 Gb) of the porcine genome.

Grouped by their corresponding categories of QTLs, the enriched traits contained 54 meat and carcass traits, 22 health traits, 6 exterior traits, 5 production traits and 2 reproduction traits. To adjust for any bias due to uneven distribution of the total number of QTL categories in the Animal Genome PigQTLdb (release 32), the frequencies of the QTL categories was calculated as the number of enriched traits in the summarised category divided by total number of the corresponding summarised category from database. The trait category with highest adjusted frequency was meat and carcass with 0.004 and the lowest was reproduction with 0.001 (Table 5). All categories were constructed and named by the Animal Genome PigQTLdb database [22] and not by the authors of this study.

**Table 5:** Summary of categories from the enriched QTL traits.

Trait category	Number of enriched traits	Total number of QTLs in category (December 2017)	Adjusted number of enriched traits
Meat and carcass	54	13,392	0.004
Health	22	6,011	0.004
Production	5	1,875	0.003
Exterior	6	2,366	0.003
Reproduction	2	1,966	0.001

Categories of enriched traits significantly overlapped by filtered *cis*-acting eQTLs. Total number of traits in categories was obtained from Animal Genome PigQTLdb (release 32) in December 2017. Adjusted number of enriched traits was calculated as the number of enriched traits divided by total number of the traits in the category.

A total of four boar taint relevant QTL traits were enriched: “Fat androstenone level", "indole, laboratory", "Off-Flavor Score" and "Overall impression, sensory panel". The traits were significantly overlapped by a total of 35 filtered *cis*-acting eQTLs associated with the genes *ARMC7*, *BAIAP2*, *TEN1*, *HEXDC*, *INTS2*, *ATP13A3*, *ENSSSCG00000004117*, *ENSSSCG00000024047*, *AOC2*, *MAPT*, *EPHA3*, *PLCD1*, *BBOX1*, *KALRN*, *NAALADL1*, *ENSSSCG00000029252* an*d ENSSSCG00000011817*. Full results table from QTL trait enrichment test is available in S5 File.

#### Candidate eQTLs

Identification of candidate eQTLs for gene-based selection was performed by analysing the filtered *cis*-acting single- and multi-tissue eQTLs for genotypes with lowest EBVs for boar taint. The criteria for candidacy was defined on two parameters: i) a filtered *cis*-acting eQTL containing a genotype with a median of summarised EBV, equal to or lower than the maximum summarised EBV of the low boar taint pigs (EBV_max_ = -0.0699208) and ii) significantly overlapping a known boar taint relevant QTL trait by its genomic coordinates. A total of 35 filtered *cis*-acting eQTLs associated with 17 unique genes with low EBVs for skatole and human nose score were selected as candidate eQTLs for gene-based selection (Table 6). Full results table of the candidate eQTLs are available in S6 File.

**Table 6:** Candidate *cis*-acting eQTLs for gene-based selection.

SNP	Gene	Candidate genotype	Mean EBVs genotype	FDR-EBV	Tissue	Sign. overlapping known QTL trait
rs81342651	*BAIAP2*	CC	-0.732	0.029	liver	Fat androstenone level
rs81265837	*ARMC7*	AA	-0.696	0.000	both	Fat androstenone level
rs81435284	*ARMC7*	AA	-0.696	0.000	both	Fat androstenone level
rs81441228	*EPHA3*	GG	-0.412	0.040	testis	Fat androstenone level
rs80817135	*KALRN*	CC	-0.805	0.045	testis	Fat androstenone level
rs81448091	*ENSSSCG00000029252*	AA	-0.805	0.048	testis	Fat androstenone level
rs81357053	*BBOX1*	AA	-0.412	0.044	liver	Fat androstenone level
rs81305789	*BBOX1*	AA	-0.412	0.044	liver	Fat androstenone level
rs81340851	*NAALADL1*	TC	-0.412	0.045	liver	Fat androstenone level
rs81212188	*NAALADL1*	AG	-0.412	0.045	liver	Fat androstenone level
rs81359616	*NAALADL1*	TC	-0.412	0.045	liver	Fat androstenone level
rs81309447	*ATP13A3*	AG	-0.248	0.020	liver	Off-Flavor Score
rs81478384	*ATP13A3*	TT	-0.805	0.020	liver	Off-Flavor Score
rs81303987	*ATP13A3*	AC	-0.248	0.020	liver	Off-Flavor Score
rs80803537	*PLCD1*	AG	-0.499	0.042	liver	Off-Flavor Score
rs81448091	*ENSSSCG00000011817*	AA	-0.805	0.048	testis	Off-Flavor Score
rs80919384	*ENSSSCG00000024047*	TC	-0.412	0.028	liver	Off-Flavor Score
rs337275357	*ENSSSCG00000024047*	AG	-0.412	0.028	liver	Off-Flavor Score
rs81433762	*TEN1*	AA	-0.805	0.006	liver	Overall impression, sensory panel
rs81434107	*TEN1*	AA	-0.805	0.006	liver	Overall impression, sensory panel
rs81311681	*HEXDC*	TT	-0.768	0.013	liver	Overall impression, sensory panel
rs81293055	*ENSSSCG00000004117*	AG	-0.294	0.025	testis	indole, laboratory
rs80806865	*ENSSSCG00000004117*	AG	-0.294	0.025	testis	indole, laboratory
rs81305046	*BAIAP2*	AA	-0.412	0.002	liver	indole, laboratory
rs81438833	*BAIAP2*	CC	-0.412	0.002	liver	indole, laboratory
rs81439307	*BAIAP2*	CC	-0.412	0.002	liver	indole, laboratory
rs81440749	*BAIAP2*	GG	-0.412	0.002	liver	indole, laboratory
rs81434341	*HEXDC*	AA	-0.768	0.013	liver	indole, laboratory
rs81271924	*HEXDC*	TT	-0.768	0.013	liver	indole, laboratory
rs81283210	*HEXDC*	AA	-0.768	0.017	liver	indole, laboratory
rs81233143	*INTS2*	TT	-0.412	0.018	liver	indole, laboratory
rs327709564	*AOC2*	AG	-0.294	0.028	liver	indole, laboratory
rs80864895	*HEXDC*	GG	-0.732	0.029	liver	indole, laboratory
rs81238764	*MAPT*	AG	-0.412	0.029	liver	indole, laboratory
rs80864895	*BAIAP2*	GG	-0.732	0.029	liver	indole, laboratory

SNP = Single nucleotide polymorphism, Candidate genotype = The best genotype for gene-based selection associated with animals with lowest summarised EBVs for skatole and human nose scores, Mean EBVs genotype. = Mean summarised EBVs in animals with the candidate genotype, FDR-EBV = False Discovery Rate of association test with summarised estimated breeding values (EBVs) of skatole and human nose scores for the eQTL, Tissue = Tissue of eQTL identification, Sign. overlapping known QTL trait = The QTL trait which was significantly overlapped by the candidate eQTL from its genomic coordinates.

**Fig 4:**
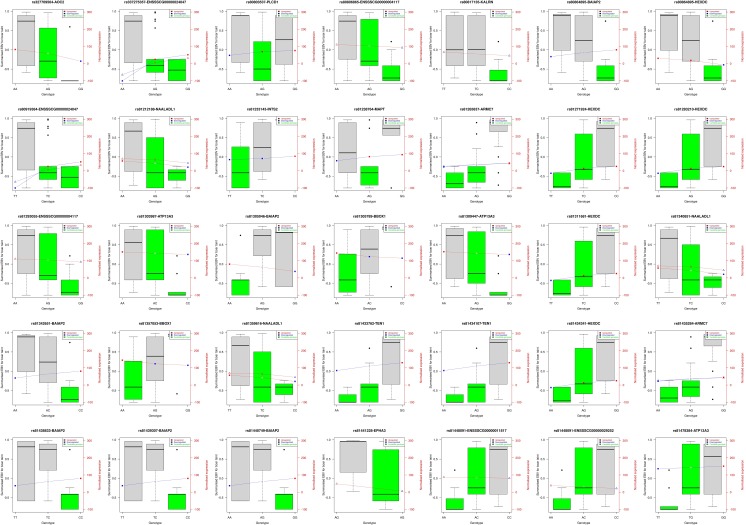
Candidate eQTLs for selection. The left Y-axis indicates summarised estimated breeding values (EBVs) for skatole and human nose score. The right Y-axis indicates the mean normalised expression levels where liver is symbolised with a square and testis is symbolised by a triangle. Due to filtering of low gene counts, some eQTLs only have expression levels from one tissue presented. The X-axis indicates the three possible genotypes of each eQTL. The title indicates the rs code of SNP and gene name associated with the eQTL. Red box indicates upregulation and blue box indicates downregulation of the eQTL at each genotype. A green barplot indicates candidate genotypes for gene-based selection, defined as genotypes with a median of summarised EBV equal or lower than the maximum summarised EBV of the low boar taint pigs.

The candidate eQTLs contained a total of 56 candidate genotypes which were distributed as follows, indicated by the gene associated with eQTLs and the number of candidate genotypes associated with the gene in parentheses: *HEXDC* (9), *ATP13A3* (6), *BAIAP2* (6), *NAALADL1* (6), *ARMC7* (4), *ENSSSCG00000004117* (4), *ENSSSCG00000024047* (4), *TEN1* (4), *AOC2* (2), *BBOX1* (2), *ENSSSCG00000011817* (2), *ENSSSCG00000029252* (2), *EPHA3* (1), *INTS2* (1), *KALRN* (1), *MAPT* (1) and *PLCD1* (1) (Fig 4).

## Discussion

### Advantages and limitations

This study applied a systems genomics approach to investigate genetic variants affecting genome-wide gene expression levels in multiple porcine tissues from pigs with known genetic merits of boar taint. We identified and functionally characterised single- and multi-tissue *cis*- and *trans*-acting expression quantitative trait loci (eQTLs) from liver and testis of Danish Landrace pigs. Subsequently, we filtered for significant association with sire-obtained estimated breeding values (EBVs) of skatole and human nose scores (HNS) and gene expression profiles of liver and testis obtained from each animal. Hence, we obtained only the most relevant eQTLs (filtered eQTLs) containing genotypes with actual association with levels of summarised EBVs. These eQTLs were subjected to functional characterisation of enriched functions and pathways and tested for enrichment of known QTL traits curated by the global Pig Quantitative Trait Locus (QTL) Database (PigQTLdb) [22]. The enrichment test revealed filtered *cis*-acting eQTLs with significant genomic overlap of boar taint QTL traits. These eQTLs were extracted and designated as candidate eQTLs. Furthermore, the enrichment test revealed potential QTL traits that could be affected by gene-based selection performed with genetic markers residing on or near the loci of the eQTLs. To the best of our knowledge, this is the first study of its kind to report candidate eQTLs associated with EBVs for skatole and HNS. The results indicate actual SNP genotypes for the identification of animals with low EBVs with respect to two important boar taint parameters with the potential for development of an inexpensive and rapid DNA test.

A limitation of the study is related to the definition of a *cis*- and *trans*-acting eQTL. The threshold of the *cis*-distance was obtained from a previous study on obesity that was conducted in the porcine genome [33]. In the aforementioned study, the authors defined a *cis*-acting eQTL as having a threshold distance of 1 Mb between gene and SNP. The threshold was calculated based on genome comparisons among species [33]. Hence, we used the same physical distance of 1 Mb as a threshold to define the *cis* -acting eQTLs. Ideally, the definition of a local (*cis*-acting) eQTL should also consider the linkage disequilibrium (LD) patterns and the persistence of LD phase in the animal model used in a study. LD structure has been estimated for a variety of pig breeds, e.g., US and Finnish breeds [61, 62]. Knowledge regarding LD structure is important in performing genetic mapping on economically important traits because gene-based selection relies on the LD between causative variants and genetic markers [63]. Furthermore, examining the persistence of LD phase in breeds is also important. The persistence of phase is a measure of consistency of LD phase for pairs of SNPs between two populations [61]. Persistence of phase explains why linkage between a marker and a QTL may differ between populations [64]. In a recent study on three Danish pig breeds (Duroc, Landrace and Yorkshire), the authors found local patterns of LD to vary between breeds because of “old inbreeding” of Duroc, but the three breeds showed similar patterns of LD on the chromosomal level [65]. In this study, we filtered for LD structure as part of genotype filtering, but ideally, the examination of LD should be included in the definition of a local *cis*-acting eQTL. However, we provided several layers of stringent eQTL filtering downstream of the eQTL identification, which should counter any breed-specific differences in LD patterns.

### General findings

#### Tissue differences

Before filtering, the number of eQTLs in testis was found to be twice the number of eQTLs in liver (*n* = 5,879 *vs*. *n* = 2,800). However, by filtering of the eQTLs for association with EBVs for skatole and HNS, the number of eQTLs was reduced ten-fold, and the differences between tissues were relatively small. Interestingly, liver then had a larger number of eQTLs (*n* = 205) than testis (*n* = 109). The reason for the differences between tissues is unclear. The number of mapped reads was nearly the same across the two tissues (7.76 *vs* 7.04 million reads in liver and testis, respectively). The numbers of mapped reads were mostly similar to those reported in previous research using RNA-Seq on porcine tissue, ranging from 8.5 to 9.1 million reads [66, 67]. In another study on candidate genes of boar taint, tissue differences between differentially expressed genes (DEG) were also found but with more DEG in testis than in liver (*n* = 46 *vs*. *n* = 25) [68]; however, identification of DEG cannot be compared with association of gene expression with genotypes. The disparity between the tissues in our study is likely related to technical differences in sample preparation, as both tissues were prepared using punch biopsies at the slaughter line. Finally, we did not use the raw eQTLs for analysis, for which the numeric disparity was nearly double, but only the eQTLs with significant association with the EBVs where the disparity was close to negligible.

#### Chromosomal densities of eQTLs

The densities of unfiltered eQTLs were highest on pig chromosomes SSC6, SSC14, SSC12, SSC4 and SSC7, in descending order. In comparing the densities of filtered eQTLs, SSC12, SSC1, SSC13, SSC9 and SSC14 showed the highest densities. The chromosomes with the highest densities of unfiltered eQTLs should be interpreted as chromosomes with a high prevalence of regulating loci affecting many traits and not only boar taint. The results are indeed interesting within candidate biomarker research. It appears that SSC6 and SSC14 are heavily inhabited by eQTLs, which could be possible hubs of regulatory control of a multitude of genes. In the context of boar taint, SSC1 and SSC14 had the highest densities of filtered eQTLs. Interestingly, previous literature employing genome-wide association study (GWAS) on Danish Landrace boars identified SNPs with significant association with skatole levels on mainly SSC14 and SSC6 [15]. In a similar GWAS study on commercial Duroc-based boars, SSC1 and SSC6 were found to contain major genetic factors that affected androstenone levels [13]. Identification of QTLs associated with skatole and indole in crossbred pigs (Meishan and Landrace) revealed the highest frequency of QTLs to be located on SSC14. Furthermore, the authors found QTLs affecting androstenone levels on SSC2, SS4, SSC6, SSC7 and SSC9, with SSC6 being the location of the QTL that causes the unpleasant “boar taste” of meat [11]. Another study located the QTLs for androstenone levels in fat tissue on SSC3, SSC4, SSC7, SSC14 and the ends of short arms of SSC6 and SSC9 [10]. A third study found SSC6 as the location for skatole levels but found no QTLs for androstenone levels, likely because of the lack of sexual maturity in the sampled pigs [25]. In conclusion, previous studies found loci affecting skatole and androstenone levels located mainly on SSC14 and SSC6. Thus, a high degree of similarity was evident between locations of known loci associated with boar taint and our findings on chromosomal densities of filtered eQTLs. Although we did not use EBVs for androstenone, previous research has documented a high correlation between HNS phenotype and androstenone levels [69], which might have contributed to the similarity. We cannot conclude that EBVs of skatole and HNS are mainly regulated by SSC14, as this chromosome also showed the highest densities of unfiltered eQTLs, which introduce a bias into the results. However, it appears reasonable to confirm that SSC14 (as well as SSC1) is associated with important loci controlling the development of boar taint.

### Analysis of eQTLs

#### Liver

In liver, we identified *CYP2R1* to be associated with the filtered eQTLs. *CYP2R1* encodes a member of cytochrome P450 superfamily (CYP) enzymes. These enzymes are known to catalyse many reactions involved in the synthesis of cholesterols, steroids and lipids and many have direct implications for the development of boar taint (as reviewed by Rasmussen and Zamaratskaia [70]). Specifically, *CYP2R1* encodes a vitamin D 25-hydroxylase participating in the synthesis of active vitamin D [71] and has been listed as a candidate gene for boar taint [16]. Furthermore, previous research from our group on the same animals identified *CYP2R1* as a differentially expressed gene in testis [41]. Our findings suggest that eQTLs regulating *CYP2R1* could play important roles in the development of boar taint, and the gene should be the subject of further research. Other CYP Family 4 members were also found to be associated with the filtered eQTLs: *CYP4A22* in *cis*-acting eQTLs from liver and *CYP4F11* in *trans*-acting eQTLs from testis. Both CYPs of this family encode monooxygenases involved in drug metabolism and synthesis of cholesterol, steroids and other lipids [72, 73]. In addition, *CYP4V2* was found to be a differentially expressed genes in testis [41]. In conclusion, CYPs are a particularly interesting group of enzymes, but none were found as candidate eQTLs with significant overlap with boar taint QTL traits. Additional testing is needed to confirm a relationship between the low-EBV genotypes of the CYP eQTLs and relevant boar taint traits.

*GSTO1* and *GSTA4* encode a member of glutathione S-transferases (GSTs) and were found to be associated with the filtered eQTLs. GSTs are known for their role in the catalysis of conjugation reactions in endogenous substances, haeme, fatty acids, xenobiotics and products of oxidative processes [74]. Previous work by our group on the same animals found *GSTO1* as a candidate biomarker in liver [41]. A close relative, *GSTO2*, has been speculated to be involved in excretion of skatole from the porcine body [75]. Both *GSTO1* and *GSTA4* showed downregulation in the high-boar-taint group in liver. This pattern could be explained by decreased excretion of boar taint compounds such as skatole, which would be consistent with previous findings pertaining to *GSTO1* in liver [76]. Interestingly, we found that *GSTO1* and *GSTA4* were highly upregulated in testis, which was consistent with previous findings on *GSTO1* in Duroc pigs [77]. In this study, the authors hypothesised that the upregulation of *GSTO1* and GSTs in general in the testis was caused by the biological role of GTSs as transporters of intracellular steroids to their site of action [74]. The authors also found breed-specific expression patterns of GSTs. Finally, we found eQTLs associated with *GSTO1* to enrich a number of QTL traits related to growth and health parameters, including backfat thickness, feed intake, ham weight and linolenic acid content. Hence, selection using eQTLs associated with the gene should be monitored carefully.

#### Testis

In testis, *RCC1L* was represented within the filtered *cis*-acting eQTLs. *RCC1L* encodes a protein associated with a regulator of chromosome condensation 1-like repeats and may function as a guanine nucleotide exchange factor [78]. Recently, the protein was found to be an essential component in oxidative phosphorylation, in which it forms a 16S rRNA regulatory module [79]. Previous studies have found an association between oxidative phosphorylation and boar taint in the testis of pigs [80, 81]. In the current study, we found eQTLs associated with *RCC1L* to enrich a number of QTL traits, including positive enrichments for “Days to 105 kg”, “Conductivity 45 minutes post-mortem” and “Carcass temperature (24 hr post-mortem)”. Because of the lack of previous knowledge on the subject and significant genomic overlaps with known boar taint QTL traits, it is not clear whether the eQTLs associated with *RCC1L* are actually associated with boar taint. However, the eQTLs are located in genomic loci where important production traits also reside, which represent interesting results and motivation for further research.

*UROS* encodes an enzyme that catalyses a step in porphyrin biosynthesis in the haeme biosynthetic pathway. Porphyrins are cofactors for a multitude of enzymes with a diverse range of roles, such as methionine synthesis (vitamin B12) or oxygen transport (haeme). Deficiency in enzyme activity because of autosomal recessive inheritance can lead to congenital erythropoietic porphyria (CEP) [82]. It is not clear why *UROS* was associated with filtered eQTLs in liver and testis. The eQTLs associated with *UROS* enriched known QTL traits such as “Base excess”, “Creatinine level”, “Loin muscle area” and “Vertebra number”. Interestingly, expression of *UROS* was found to be co-expressed by correlation (R^2^ > 0.5) with the expression profiles of four other genes. However, the occurrence of *UROS* appears strange, and to our knowledge, no biological relationship with boar taint is currently evident.

#### Functional characterisation

Functional characterisation of the filtered *cis*-acting eQTLs identified from liver revealed association with androgen and oestradiol receptor signalling and regulation. The eQTLs associated with these functions contained the genes *SFRP1*, *ESR1*, *MAPT* and *RNF14*. *SFRP1* encodes an inhibitor of the Wnt/β-catenin pathway. Previous research concluded that Wnt/β-catenin signalling is necessary for the development and masculinisation of male genitalia [83]. Furthermore, Wnt/β-catenin has been found to be associated with proliferation and self-renewal of mouse and human spermatogonia [84]. Wnt/β-catenin signalling is regulated by a decrease in expression of Wnt inhibitors, such as *DKK2* and *SFRP1* [83]. In this study, *SFRP1* was found to be downregulated in the high-boar-taint group in liver. This finding was expected as androgen signalling leads to decreased expression of Wnt/β-catenin inhibitors. Oddly, the gene was associated with a filtered eQTL in liver but not in testis. We speculate that increased androstenone is associated with an increased level of androgen signalling and, in turn, a decrease in the general tissue expression of *SFRP1*. However, this relationship does not explain the lack of finding an eQTL associated with the gene in testis, where spermatogonia are developed. Furthermore, the Wnt inhibitor *DKK2* was recently found to be the highest upregulated gene in testis of high androstenone pigs [68], in contrast with the expression pattern of *SFRP1* found in this study. Finally, eQTLs associated with *SFRP1* were found to enrich known QTL traits of two important muscle fibre characteristics: “Muscle moisture percentage” and “Semimembranosus angle”. Because of the ambiguous results, more research is needed on these important Wnt inhibitors and their relationship with boar taint development and meat quality. *ESR1* encodes an oestrogen receptor that binds steroid hormones and was found to be associated with *cis*-acting eQTLs from testis. Oestrogen is secreted in large amounts from the testis [85], and concentrations have been found to be correlated with androstenone in adipose tissue [86, 87]. Interestingly, eQTLs associated with *ESR1* enriched a number of QTL traits for muscle, carcass and the fertility trait of teat numbers but no overlapping boar taint QTL traits. Finally, *RNF14* encodes a RING zinc finger that interacts with the androgen receptor and may function as a coactivator and transcriptional regulator [88]. *RNF14* was found to play an important role in the regulation of mitochondrial and immune-related genes in skeletal muscle [89]. The eQTLs associated with the gene enriched five important production and muscling QTL traits, including “Cortisol level variations” and “Potassium level”. To our knowledge, no research has provided a link between *RNF14* and boar taint, but an association is highly likely because of the gene’s regulation of androgen receptors.

#### Enrichment test for possible selection effects

An enrichment test to reveal enriched known QTL traits was performed to identify possible selection effects using the filtered *cis*-acting eQTLs and to identify candidate eQTLs. The test revealed 89 enriched traits, which were mainly related to meat and carcass features and production. The results provide an approximation of which traits may be affected if gene-based selection is performed on the genomic coordinates of the *cis*-acting eQTLs. Two enriched traits were in the reproduction category: “Left teat number” and “Litter size”. The traits were enriched by eQTLs associated with a total of 30 unique genes, including six genes intersecting with the candidate eQTLs: *INTS2*, *AOC2*, *HEXDC*, *BBOX1*, *KALRN* and *EPHA3*. A common concern regarding selection for lowered levels of boar taint compounds is negative consequences on male fertility [90]. Androstenone is synthesised from pregnenolone during sexual maturity together with other testicular steroids, such as testosterone and oestrogens [91, 92]. These have been established to be important sex steroids affecting male fertility [93, 94]. An unfavourable genetic correlation between androstenone levels and sex steroids was reported [18]. Nevertheless, the possibility of selection for reduced boar taint was found to be a viable solution in a large-scale genetic study involving direct measures of fertility and concentrations of boar taint compounds [90]. We speculate that our finding of two enriched traits for reproduction would confirm the viability of selection with little chance of a negative effect on fertility if one applies careful monitoring. However, we did find several eQTLs in liver and testis associated with *SFRP1*, which is related to male reproduction and fertility. Thus, future studies should focus on the roles of these genes and whether any causal relationship between them and male fertility exists. To the authors’ knowledge, we are the first to report eQTLs associated with EBVs of skatole and HNS in Danish Landrace pigs and known QTL traits that could possibly be affected by selection on the filtered eQTLs or loci/genes in their vicinity. However, it should be clearly noted that the findings of the QTL enrichment test are highly speculative, and no experimental testing, other than a statistical association test, has been performed to confirm the associations. Furthermore, the QTL traits are obtained from a large number of publications that differ in their methodologies. Finally, the QTL regions curated by the Animal Genome PigQTL db database are very large, likely because of the difficulty of translating cM into genomic coordinates in bp, which is confirmed in the disclaimer on the Animal Genome website. Validation and verifications must be performed when these data are used as a basis for further research.

#### Candidate eQTLs for selection of low EBV animals

A total of 35 candidate eQTLs were identified from the filtered *cis*-acting eQTLs, which were selected based on the criteria of significant association with EBVs and genomic overlap with boar taint QTL traits. The candidate eQTLs were sorted according to the number of candidate genotypes for low EBVs (displayed in parentheses): *HEXDC* (9), *ATP13A3* (6), *BAIAP2* (6), *NAALADL1* (6), *ARMC7* (4), *ENSSSCG00000004117* (4), *ENSSSCG00000024047* (4), *TEN1* (4), *AOC2* (2), *BBOX1* (2), *ENSSSCG00000011817* (2), *ENSSSCG00000029252* (2), *EPHA3* (1), *INTS2* (1), *KALRN* (1), *MAPT* (1) and *PLCD1* (1). The candidate eQTLs associated with *HEXDC* comprised the largest number of candidate genotypes. *HEXDC* encodes a hexosaminidase involved in metabolism and glycan degradation. These enzymes have been shown to play key roles in cellular physiology and health and are also considered essential for the development of mammals [95]. More importantly, hexoaminidases are present in very high concentrations in the epididymis of the male pig reproductive organs [96] where hexosaminidases have been described as key players in post-testicular sperm maturation [97]. In this study, we found eQTLs associated with *HEXDC* to enrich two both boar-taint-related QTL traits (“Overall impression, sensory panel” and “indole, laboratory”) but also the fertility trait “Left teat number”. Hence, it appears plausible to assume that the gene is associated, to a degree, with fertility; interestingly, however, the eQTLs associated with the genes were identified in the liver. The gene was upregulated with increasing EBVs, which could indicate a correlation between hexoaminidases and steroid hormone production. However, further validation in large trials should be performed to assess the role of the gene with respect to the boar taint condition and fertility. *ATP13A3* encodes a P-type ATPase family that transports cations across membranes [98]. The gene has been analysed for its potential as a biomarker for early detection of pancreatic cancer [99]. In the context of boar taint, other ATPases involved in ATP synthesis have been reported as candidate genes in boar taint development [75, 100], but no data are available for *ATP13A3*. However, the eQTLs associated with *ATP13A3* enrich a total of 24 QTL traits on many different parameters including boar-taint-relevant QTLs. It could be speculated that the gene is somehow affected by increased skatole metabolism, as the level of gene expression is clearly correlated with high- and low-EBV genotypes. *ARMC7* encodes an essential nuclear protein and has been found to be amplified in several cancer tissues and cell lines [101]. Within cancer research, *ARMC7* has been the subject of research as a prognostic marker for breast cancer [102], but to our knowledge, no studies have shown a relationship between the gene and boar taint. Interestingly, the gene was markedly upregulated in high-EBV genotypes and vice versa in low-EBV genotypes, implying association with boar taint. Furthermore, the eQTLs associated with *ARMC7* enriched known QTL traits for boar taint, such as “Fat androstenone level” and “indole, laboratory”. However, the eQTLs also enriched a number of important meat quality, production and growth traits, such as “Juiciness score”, “Conductivity 45 minutes post-mortem” and “Inside ham weight”. Hence, it is plausible to assume that the eQTLs are located within QTL-rich genomic areas of SSC12. Careful monitoring of the enriched traits should be performed if selection is performed on this subset of eQTLs. Finally, *AOC2* encodes a copper amine oxidase that catalyses oxidative conversion of amines to aldehydes and ammonia. The eQTLs associated with *AOC2* were found to enrich a QTL trait for indole concentrations but also enrich the reproductive trait of “Left teat number”. Interestingly, the enzyme relative of aldehyde oxidase (AO) was considered negatively correlated with levels of skatole in fat because of its role in skatole metabolism in liver [103]. The authors proposed that a high hepatic enzyme activity of AO was associated with low levels of skatole, but other reports found AO to be uncorrelated with skatole in plasma and fat [104]. Interestingly, our findings show a contrary pattern of gene expression pattern proposed for AO as *AOC2* is upregulated with high EBVs for skatole and HNS. This finding could be explained by an activating effect of skatole as a xenobiotic compound, but further research should be performed to investigate the role of *AOC2* in hepatic clearance of skatole.

## Conclusions

This study applied an integrative systems genomics approach to identify and characterise single-tissue eQTLs associated with EBVs for skatole and HNS and with a number of known genes, such as *CYP2R1*, *GSTO1*, *GSTA4*, *RCC1L*, *UROS*, *SFRP1* and *RNF14*. Furthermore, the study provided descriptive statistics of densities of eQTLs on chromosomes of Danish Landrace pigs, among which SSC1, SSC12 and SSC14 were confirmed as the main chromosomes of boar taint regulation. Using a QTL enrichment test, we identified 89 known QTL traits to be enriched from the genomic coordinates of the filtered eQTLs. The enriched traits included important meat and carcass, production, exterior and reproduction traits. Any selection process must closely monitor both positive and negative effects on these traits, which included muscle, fat and weight gain. Furthermore, reproduction-associated eQTLs were found, which warrants further consideration of careful monitoring of any consequences for fertility in future breeding schemes. From four enriched boar taint QTL traits, a total of 35 filtered *cis*-acting eQTLs with genotypes for low EBVs of skatole and HNS were extracted and designated as candidate eQTLs. The candidate eQTLs are an interesting subset of readily available genes and SNPs with strong association with lowered EBVs and genomic locations in confirmed boar taint regions. Future work should focus on the validation of the candidate eQTLs in large populations as well as in breeds other than Danish Landrace pigs to develop a sensitive and specific DNA test for optimised gene-based animal breeding under industrial settings.

## Supporting information

S1 File Illustration of the study design. Liver, testis and ham muscle was obtained from non-castrated Danish Landrace male pigs. The liver and testis was subjected to RNA extraction and RNA-sequencing (RNA-Seq) to obtain gene expression profiles. DNA was extracted from ham muscle and subjected to genotyping by Illumina Porcine 60K SNP-chip. The software Matrix eQTL identified single- and multi-tissue eQTLs which were subsequently filtered by a statistical model comparing EBVs and expression profiles from animals grouped by the three genotypes available of each eQTL. The filtered single- and multi-tissue eQTLs were subjected to functional characterisation and a QTL trait enrichment test to find potential known QTL traits that could be affected by selection. Finally, eQTLs enriching boar taint QTL traits from PigQTLdb by significant (FDR < 0.05) genomic overlaps were identified and evaluated as candidate eQTLs for future biomarker development.
(TIF)

S2 File Spreadsheet of single-tissue the filtered eQTLs of liver and testis. Result table obtained from analysis of eQTLs with significant (FDR < 0.05) association with the summarised estimated breeding values of skatole and human nose scores.
(XLS)

S3 File List of GO terms and KEGG pathways enriched in filtered eQTLs from liver and testis. Result table obtained from functional characterisation of liver and testis eQTLs with significant (FDR < 0.05) association with the summarised estimated breeding values of skatole and human nose scores. Gene symbols are included for each GO term and KEGG pathway.
(XLS)

S4 File Spreadsheet of the filtered multi-tissue eQTLs. Result table obtained from analysis of multi-tissue eQTLs with significant (FDR < 0.05) association with the summarised estimated breeding values of skatole and human nose scores.
(XLS)

S5 File QTL traits enriched by the filtered multi-tissue eQTLs. Result table obtained from enrichment test with using filtered *cis*-acting eQTLs on known QTL traits from Animal Genome PigQTLdb. The test revealed QTL traits which were significantly (FDR < 0.05) overlapped by the eQTLs and the genes associated with the eQTLs that overlapped the QTL traits.
(XLS)

S6 File Spreadsheet of the candidate eQTLs. The candidate eQTLs were defined as filtered *cis*-acting eQTLs which enriched known boar taint QTL traits from the PigQTLdb. The candidate eQTLs could be useful in future development of biomarkers for gene-based selection schemes of pigs with lowered boar taint. The spreadsheet includes rs codes for SNPs, gene names and the genotypes associated with lowest values of summarised estimated breeding values for skatole and human nose scores.
(XLS)
