# Supplementary figures and images for: Systems genomics study reveals expression quantitative trait loci, regulator genes and pathways associated with boar taint in pigs
Source: PLoS One. 2018 Feb 13;13(2):e0192673. doi: 10.1371/journal.pone.0192673 (PMC5811030; doi:10.1371/journal.pone.0192673)

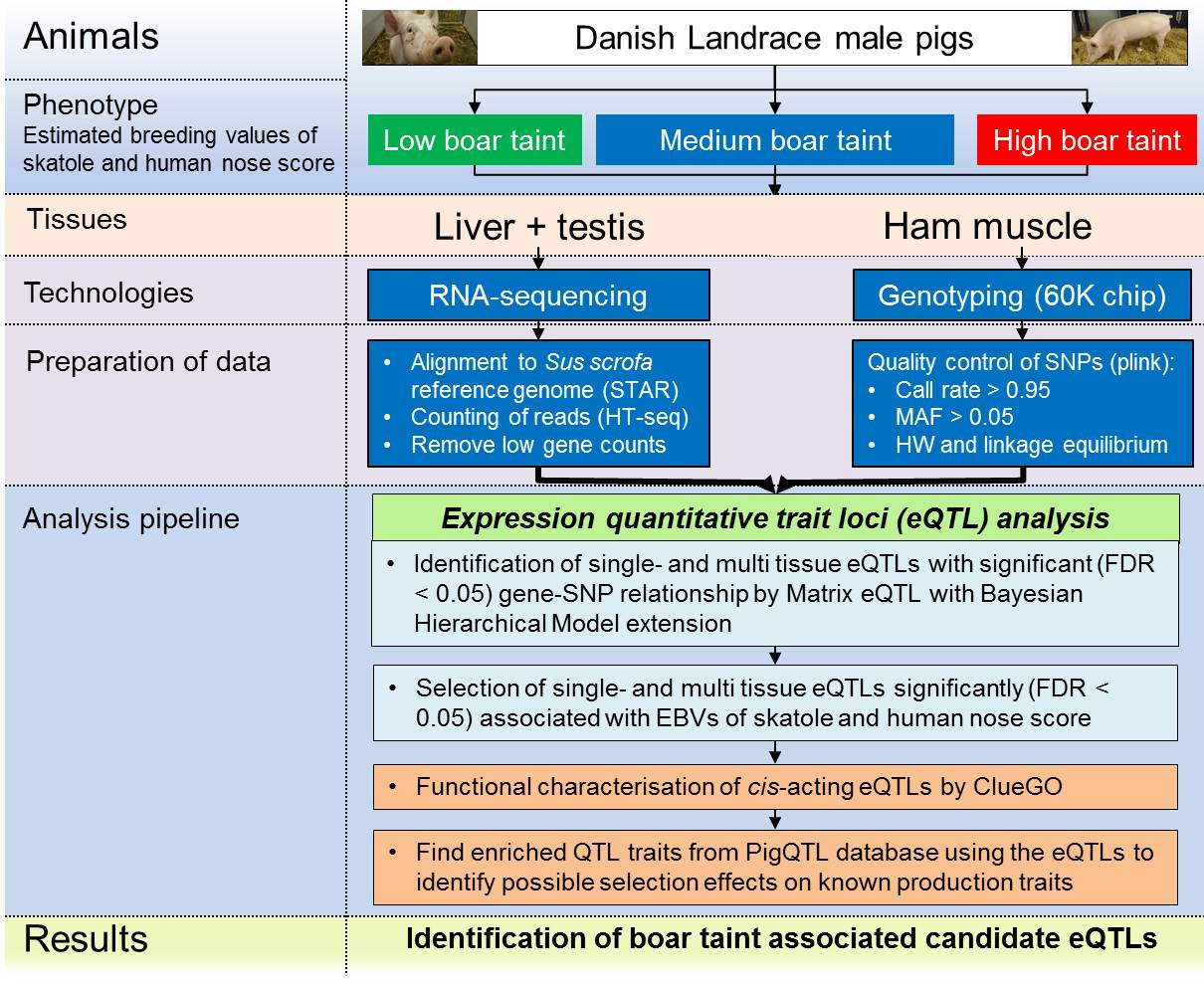

Supplement: S1 File — Liver, testis and ham muscle was obtained from non-castrated Danish Landrace male pigs. The liver and testis was subjected to RNA extraction and RNA-sequencing (RNA-Seq) to obtain gene expression profiles. DNA was extracted from ham muscle and subjected to genotyping by Illumina Porcine 60K SNP-chip. The software Matrix eQTL identified single- and multi-tissue eQTLs which were subsequently filtered by a statistical model comparing EBVs and expression profiles from animals grouped by the three genotypes available of each eQTL. The filtered single- and multi-tissue eQTLs were subjected to functional characterisation and a QTL trait enrichment test to find potential known QTL traits that could be affected by selection. Finally, eQTLs enriching boar taint QTL traits from PigQTLdb by significant (FDR < 0.05) genomic overlaps were identified and evaluated as candidate eQTLs for future biomarker development. (TIF) [file pone.0192673.s001.tif]
